# Dietary Nitrate Lowers Blood Pressure: Epidemiological, Pre-clinical Experimental and Clinical Trial Evidence

**DOI:** 10.1007/s11906-015-0623-4

**Published:** 2016-01-27

**Authors:** Lorna C. Gee, Amrita Ahluwalia

**Affiliations:** William Harvey Research Institute, Barts & The London School of Medicine & Dentistry, Queen Mary University of London, Charterhouse Square, London, EC1M 6BQ UK

**Keywords:** Nitric oxide, Nitrate, Nitrite, Animal models, Nutrition

## Abstract

Nitric oxide (NO), a potent vasodilator critical in maintaining vascular homeostasis, can reduce blood pressure in vivo. Loss of constitutive NO generation, for example as a result of endothelial dysfunction, occurs in many pathological conditions, including hypertension, and contributes to disease pathology. Attempts to therapeutically deliver NO via organic nitrates (e.g. glyceryl trinitrate, GTN) to reduce blood pressure in hypertensives have been largely unsuccessful. However, in recent years inorganic (or ‘dietary’) nitrate has been identified as a potential solution for NO delivery through its sequential chemical reduction via the enterosalivary circuit. With dietary nitrate found in abundance in vegetables this review discusses epidemiological, pre-clinical and clinical data supporting the idea that dietary nitrate could represent a cheap and effective dietary intervention capable of reducing blood pressure and thereby improving cardiovascular health.

## Introduction

### The Burden of Hypertension

Cardiovascular disease is a significant disease burden worldwide, responsible for approximately 17.5 million (31 %) deaths each year according to the World Health Organization (WHO) [1]. Hypertension particularly has been estimated to account for nearly 10 million of these deaths, and is now considered the leading risk factor for global disease burden [2]. The annual economic impact of hypertension in the US alone (through both direct and indirect costs) has been estimated to be $46.4 billion, and by 2030 is predicted to rise to nearer $274 billion [3]. Recent evidence, from both primary and secondary care electronic health records in the UK, supports the view of blood pressure as a continuum with increasing risk of cardiovascular disease with increasing pressures [4]. In particular this study suggested that perhaps current guidelines for target blood pressures are set too modestly. The study demonstrated that those with hypertension (i.e. blood pressures of ≥140/90 mmHg) at the age of 30 developed cardiovascular disease 5 years earlier than normotensive counterparts. In addition they demonstrated that, as expected, for many cardiovascular diseases a linear association with blood pressure is evident. However, perhaps unexpectedly, it was identified that in individuals with systolic blood pressures of 90–114 mmHg compared to 115–129 mmHg, disease risk continued to be substantially lower. Importantly, the SPRINT [5] (NCT 01206062) study in the US, looking at more aggressive blood pressure lowering in the over 50s to systolic pressures of 120 mmHg, has demonstrated reduced rates of cardiovascular events as compared to lowering of systolic pressures to 140 mmHg (current target blood pressure according to guidelines) including ∼1/3 reduced risk of heart attack, heart failure and stroke and reduced risk of death by ∼1/4. Such observations provide support for early and intensive blood pressure intervention and highlight a rationale for further active blood pressure lowering approaches to be adopted even by those classified according to current norms as having ‘healthy’ blood pressure.

Roughly 80 million (1 in 3) adults in the US are hypertensive, and in 2011 only 54 % of these individuals were estimated to have the condition under control [3]. In total, 83 % were aware of their hypertension, but only 77 % were being actively treated. As both hypertension (≥140/90 mmHg) and even severe hypertension (≥180/110 mmHg) are often asymptomatic [6] it is not entirely surprising that the remaining 17 % had no knowledge of their condition [3], a pattern also observed in Canada and England [7].

Thus, taking all of the above into account there is a continued imperative to find new interventions that are not only acceptable for the relatively ‘well’ population but also interventions for those where poly-pharmacy is not the preferred approach.

### A Drive for Dietary Intervention in Primary Prevention and Treatment of Rising Blood Pressure

First line treatment for patients with raised blood pressures, but not at levels appropriate for pharmacotherapy, is lifestyle modification. However, such an approach becomes perhaps more important when one considers the substantial benefits accrued, in terms of cardiovascular disease-free years of life, when reducing blood pressure well below conventional accepted levels of ‘good’ blood pressure i.e. 90–114 mmHg of systolic pressure versus 115–129 mmHg [4]. Interventions that are not drug-based are perceived as being more acceptable for patients, particularly those that are ‘symptomless’. Furthermore, with the burden of uncontrolled, untreated and undiagnosed hypertension rising in low and middle income countries [1, 8], effective interventions offering a cheap and universally acceptable treatment, resulting in improved cardiovascular health worldwide, are desirable.

Numerous studies have demonstrated the benefits of individual lifestyle measures upon blood pressure including, and of particular relevance to this review, diet. For some dietary constituents our understanding of the impact and mechanisms of action of these interventions is advanced, whilst in contrast the impact of others is more controversial. As an example salt reduction has been widely investigated with numerous and large-scale clinical trials demonstrating efficacy in blood pressure reduction [9]. These studies have resulted in policy change in numerous countries attempting to reduce salt intake and thus hypertension and cardiovascular disease burden worldwide. Another dietary intervention thought to influence blood pressure [10], that has also resulted in significant policy change is that of fruit and vegetable intake (NHS, 5 a day; US, 7 a day). However, unlike salt reduction, increasing fruit and vegetable intake has not been so easy. This is despite the fact that large-scale meta-analyses very convincingly demonstrate a decreased cardiovascular disease incidence with increased intake, with five portions of fruit and vegetables per day being the optimum level of intake [11]. In addition to the socioeconomic reasons that might underlie this [12] there is also the possibility that the considerable uncertainty and controversy with respect to mechanism of action and identification of the key element responsible for the benefits plays a role. Recently, however, there has been growing support for the view that the naturally occurring compound inorganic (or ‘dietary’) nitrate (NO_3_^−^) may play a role and it is this constituent of vegetables that is the focus of this review.

Inorganic nitrate is a key nutritional constituent of many vegetables, including spinach, beetroot and celery [13, 14]. All vegetables take up nitrate from the soil since it is critical in providing a supply of nitrogen. Importantly, those vegetables that possess large green leaves tend to extract more nitrates from the soil due to the need for growth and accordingly green leafy vegetables contain the highest levels of nitrate found in the diet [15]. This fact is of interest when one considers that of all the fruits and vegetables that confer protection over the cardiovascular system it is the green leafy vegetables that provide the greatest benefit [16]. This review will discuss some of the epidemiological evidence for significant blood pressure reduction by dietary nitrate, some of the pre-clinical evidence in animal models and prospective clinical trials testing efficacy of dietary nitrate as an intervention.

### NO Supplementation and Cardiovascular Health

The importance of the L-arginine/NO pathway in cardiovascular health has long been established, with endogenous NO production by endothelial NO synthase (eNOS) found to play a pivotal role in regulating vascular tone and blood pressure in both pre-clinical and clinical studies [17, 18]. With NO production critical in helping maintain healthy vascular homeostasis [19] and reduced endogenous NO production involved in the pathogenesis of hypertensive disorders [20] it makes sense to investigate NO supplementation as a treatment for hypertension.

In the late nineteenth century, organic sources of NO such as nitroglycerin (GTN) were used to restore NO to the vasculature. However, with rapid NO release from these compounds, vasodilatory effects were acute and had no long-term ability to reduce blood pressure. The latter being due in part to the tachyphylaxis that develops rapidly in response to organic nitrates [21, 22] and also, discovered much later, due to the fact that many of these compounds induce endothelial dysfunction per se [23]. Such characteristics have of course substantially limited the clinical utility of this class of compounds, and although the organic nitrates are still used for acute symptomatic relief in angina patients and in the acute heart failure scenario, the long-term use for blood pressure reduction is not considered a viable first-line option. There is some evidence, however, that in patients with systolic hypertension once daily low-dose regimens may be useful [24, 25]. This beneficial effect is likely due to the organic nitrate-induced improvements in arterial stiffness rather than peripheral vasodilation [26].

### The Potential of *Inorganic* Nitrate—the Enterosalivary Circuit

Until recently, endogenous inorganic nitrite (NO_2_^−^) and nitrate (NO_3_^−^) were considered primarily as oxidized, largely inactive, waste metabolites of NO. However, we now know that both anions can act as storage forms of NO (for reviews see Lundberg et al. [27] and Rathod et al. [28]) and under certain conditions are being reduced back to bioactive NO.

For plasma nitrate to be successfully converted to NO (regardless of whether the source of nitrate is from NO oxidation or through ingestion of nitrate-rich foods), we rely heavily on the ‘enterosalivary circuit’. The oral bioavailability of ingested nitrate is near 100 % [29], as nitrate can be absorbed readily across the gut wall (by as of yet unknown mechanisms) and transported in the blood plasma. It is estimated that 60–75 % of this nitrate is lost from the plasma by excretion within 48 h [30]. However, approximately 25 % of nitrate in the circulation becomes concentrated in the salivary glands through active uptake from the circulation by the sialin transporter [31]. As a result, nitrate is released into the oral cavity generating up to 10 times higher saliva concentrations than that in the plasma [32, 33]. It is within the oral cavity that nitrate comes into contact with facultative bacteria [34] which reduce nitrate to nitrite, that is then swallowed and absorbed across the gut wall, resulting in increased plasma nitrite levels [32, 35]. It is now widely accepted that disruption of the enterosalivary circuit either through antibiotic administration [36] or through collection of all saliva prior to swallowing [32, 35, 37•] prevents increases in plasma nitrite ordinarily seen following oral inorganic nitrate supplementation.

Critical for biological activity of nitrate are endogenous nitrite reductases in the circulation [38], such as XOR [39, 40], which reduce plasma nitrite further to bioactive NO. This NO is then free to exert its beneficial effects on the cardiovascular system e.g. inducing vasodilation, improving measures of arterial stiffness and thus reducing blood pressure. A great many of the beneficial effects of NO are mediated by cyclic guanosine monophosphate (cGMP) (for a recent review see Francis et al. [41]) and following inorganic nitrate supplementation, increases in this sensitive marker of NO bioactivity [42] are observed [43–45]. The hypotensive effects of dietary nitrate through this pathway have now been investigated with success in human and animal studies, findings, which to some extent, have been supported by some epidemiological data.

## Epidemiological Evidence

### Fruit and Vegetable Intake, Blood Pressure and Cardiovascular Disease Risk

Whilst there is no conclusive epidemiological evidence to date supporting dietary nitrate intake reducing blood pressure, a number of studies have shown that increased consumption of fruits and vegetables reduces systolic and diastolic blood pressure by between 3–6 and 1–3 mmHg, respectively [10, 46, 47]. This reduction is thought to underlie a substantial proportion of the reductions in both cardiovascular disease risk and mortality associated with fruit and vegetable consumption [48–50].

The beneficial effects of such a diet have been proposed to be due to a number of components, including macronutrient composition e.g. protein, fat and carbohydrate [51], antioxidants [52], fiber [53] and potassium [54, 55]. However, such propositions do not fully or sufficiently account for the changes in blood pressure observed on a fruit and vegetable-rich diet [56], and meta-analyses have even concluded there appears to be no significant benefit from supplementation with antioxidants or potassium [57, 58]. Recently however, it has been proposed that inorganic nitrate may underlie at least part of the beneficial cardiovascular effect of such a diet.

This latter proposal is of particular interest since studies have shown that the greatest protection provided by fruit and vegetables is derived from the ‘green leafy’ vegetables [16, 59]. Typically, these groups of vegetables contain the highest levels of nitrate [14, 15]. However, conclusive evidence linking the inorganic nitrate content of vegetables with reductions in cardiovascular disease risk remains elusive due to a number of significant hurdles. These hurdles have recently been debated in an NIH workshop entitled ‘Dietary Nitrate and the Epidemiology of Hypertension and Cardiovascular Disease’ that was held in Washington in September 2014 (http://www.nhlbi.nih.gov/research/reports/2014-dietary-nitrate). One particularly prominent issue highlighted in this workshop is that accurate and reliable estimates of dietary nitrate ingestion are lacking. Importantly, the nitrate content of vegetables is highly variable, being dependent on a number of different factors, not least the nitrate content of the soil where the vegetables are grown, the presence or not of nitrogen-based fertilizers and the time of the day/year of harvesting. A key recommendation made by the workshop attendees is that contemporaneous assessment of nitrate/nitrite content of the diet, with assessment of blood pressure, in large cohort and population studies should be conducted together with analysis of actual exposure through measurement of in vivo nitrate/nitrite levels in plasma, urine or saliva. Such a study would be critical in confidently assessing the possible association of nitrate ingestion with cardiovascular disease risk.

### The Effects of a Traditional Japanese Diet on Blood Pressure

There are a number of observations that support the proposals made by The NIH Workshop attendees. The traditional Japanese diet, which is typically high in inorganic nitrate from vegetables such as ta cai, Chinese cabbage and spinach [60], has been associated with lower systolic and diastolic blood pressures. On the contrary, a westernized diet in the same Japanese population was associated with higher blood pressure [61]. The effects of a traditional Japanese diet (delivering 18.8 mg/kg/day) compared to a control diet (∼equal to the ADI of 3.7 mg/kg/day) have been investigated in a clinical study expanding on this finding. The traditional Japanese diet resulted in an average decrease in mean diastolic blood pressure of 4.5 mmHg (*P* = 0.01), but had no significant effect on systolic blood pressure [60].

Importantly, in this study they demonstrated that the traditional Japanese diet significantly increased plasma and salivary nitrate compared to a control diet, from 43 to 154 μM (*P* < 0.001) and 200 to 570 μM (*P* < 0.001), respectively. Crucially, plasma nitrite was also increased from 132 to 204 nM (*P* < 0.01) [60], suggesting commensal bacteria successfully converted nitrate to nitrite via the enterosalivary circuit. Such observations perhaps provide clues to the apparent longevity and reduced cardiovascular risk in those maintaining a traditional Japanese diet [62, 63].

## Pre-clinical Experimental Evidence

Fortunately, in pre-clinical animal studies it has been demonstrated that the enterosalivary circuit of nitrate is intact. For example, in both rats and mice, supplemental feeding with inorganic nitrate increases plasma nitrite levels resulting in NO-mediated bioactivity [43, 64–66]. In addition to this, limiting intake of nitrate and nitrite results in rapid depletion of tissue nitrite levels and reductions in cGMP signaling pathways, suggesting reduced NO bioactivity [67]. Finally interference with the oral nitrate-reducing bacteria eliminates nitrate bioactivity [66].

This similarity with humans has therefore permitted investigation into the effects of nitrite and nitrate on blood pressure in animal models of hypertension including Wistar Kyoto (WKY) rats (both with and without phenylephrine induced hypertension), spontaneously hypertensive rats (SHR), ‘two-kidney 1-clip’ rats and rats subjected to unilateral nephrectomy and a chronic high-salt diet [65, 68–72]. In these models nitrate reduced mean arterial blood pressure by between ∼20 and 60 mmHg. It has also been shown that the blood pressure lowering effect of nitrate in the SHR and WKY models is XOR-dependent and can be blocked by concomitant dosing with XOR inhibitor allopurinol [70].

In addition to this, nitrate has been found to ameliorate pulmonary arterial hypertension (PAH) in mice subjected to hypoxia for 3 weeks. Not only does it significantly reduce right ventricular pressure, but also reduced left ventricular hypertrophy and vascular remodeling [43]. These effects were associated with increases in plasma and lung nitrite and cGMP, with the beneficial effects of nitrate being greatly reduced following co-dosing with allopurinol. Interestingly, the effects were also reduced in eNOS knockout mice, suggesting that in addition to XOR, eNOS may also be functioning as a nitrite reductase within the pulmonary vasculature.

Some concern has been raised with respect to the relevance of some laboratory data regarding nitrate supplementation in mice (3 mmol/kg/day) to the human scenario [73]. However, whilst a dose of 3 mmol/kg/day in rodents produces rises in circulating nitrite that are bioactive, if extrapolated to humans (∼210 mmol/day for a 70-kg individual) such doses are simply not required to get a proportional rise in plasma nitrite in humans. A single dose of 5.5 mmol in the form of beetroot juice was sufficient to see a 1.6-fold rise in plasma nitrite in humans and a 10-fold increase in plasma nitrate. In a separate study assessing the activity of potassium nitrate capsules circulating levels of nitrite were raised 1.3- and 2-fold in response to 4 and 12 mmol nitrate, respectively [44]. The exact reason for this difference between mice and humans is presently unknown, but may be due to inter-species differences in the enterosalivary circuit, for example with potential differences in bacterial nitrate reductase activity, nitrate excretion and nitrite absorption across the gut lining. It is noteworthy however, that rats may be different to mice. It has been shown that doses of nitrate that resemble those consumed by humans in a nitrate and vegetable-rich meal do effectively lower blood pressure in rats [64, 66], highlighting again clear species differences that should be taken into account with pre-clinical studies.

## Clinical Trial Evidence

### Healthy Volunteer Studies

The activity of the enterosalivary circuit in humans was first described in a clinical trial by Lundberg and Govoni when they showed that plasma nitrite significantly increases following an oral sodium nitrate load or in response to a 300-g portion of spinach [32]. This phenomenon has since been observed in a great number of studies, with participants given oral nitrate supplementation in a range of ways, including vegetables, juice, modified breads and potassium/sodium nitrate capsules (see Tables 1 and 2).

**Table 1:** The effects of dietary inorganic nitrate on blood pressure in healthy volunteer studies

Author	Participants	BP primary or secondary endpoint	Primary endpoint	Secondary endpoint	Nitrate source/dose	Placebo	Effect on BP	BP measurement method	Study design	Intervention length
Ashworth et al. [74]	Healthy (19 F) mean age 20 years	Primary	BP	Plasma nitrite/nitrate	High-nitrate vegetables	Low-nitrate vegetables	SBP −4 mmHg (*P* < 0.05), DBP (NS)	Clinic	Randomized crossover (3-week washout)	Chronic 1 week
Jovanovski et al. [75]	Healthy (11 M, 16 F) mean age 25 years	Primary	Central and brachial BP, aumentation index	n/a	13.63 mmol/day spinach soup	0.01 mmol/day asparagus soup	SBP −4.05 mmHg (*P* < 0.01), DBP −4.43 mmHg (*P* < 0.05)	Clinic	Randomized, placebo-controlled, single-blind crossover (1-week washout)	Chronic 1 week
Liu et al. [76]	Healthy (6 M, 20 F) mean age 59 years	Pimary	BP, PWV, augmentation index, artery elasticity index	Salivary nitrite/nitrate	3.55 mmol (250 g spinach)	Rice milk	<SBP (*P* < 0.001), DBP (NS)	Clinic	Randomized, placebo-controlled crossover (1-week washout)	Acute 4 h
Bahra et al. [77]	Healthy *n* = 14 (M and F) mean age 28 years	Secondary	FMD, PWV, BP, Plasma, salivary and urinary nitrite/nitrate	n/a	8 mmol KNO_3_	8 mmol KCI	SBP −4.9 mmHg (*P* < 0.05), DBP (NS)	Clinic	Randomized, palcebo-controlled, double blind, crossover (7–28-day washout)	Acute 3 h
Bondonno et al. [78]	Healthy (6 M, 24 F) mean age 47 years	Secondary	FMD	BP, plasma nitrite/nitrate	2.9 mmol (200 g spinach)	Rice milk	SBP −2.7 mmHg (*P* = 0.01), DBP (NS)	Clinic	Randomized, placebo-controlled, crossover (>7-day washout)	Acute 3 h
Coles and clifton et al. [79]	Healthy (15 M, 15 F) mean age 25 years	Primary	BP	n/a	7.5 mmol beetroot and apple juice (data provided by company)	Apple juice matched for sweetness and colour	SBP −4 to −5 mmHg (*P* < 0.05)—men only	Clinic and ABPM	Randomized, placebo-contolled, double blind crossover (2-week washout)	Acute 24 h
Hobbs et al. (study 1) [80]	Healthy (18 M) mean age 31 years	Primary	BP	Urinary nitrite/nitrate	0.05, 2.3, 5.7 and 11.4 mmol beetroot juice	Water	SBP with 2.3 mmol dose (AUC *P* < 0.006), DBP with 2.3, 5.7 and 11.4 mmol doses (AUC *P* < 0.001)	ABPM	Randomized, placebo-controlled, single-blind, crossover (7-day minimum washout)	Acute 24 h
Hobbs et al. (study 2) [80]	Healthy (14 M) mean age 21 years	Primary	BP	Urinary nitrite/nitrate	1.6–1.8 mmol beetroot enriched bread	Plain white bread	SBP (NS), DBP (AUC *P* < 0.05)	ABPM	Randomized, placebo-controlled, single-blind crossover (7-day minimum washout)	Acute 24 h
Lansley et al. [81]	Healthy, competitive (9 M) mean age 21 years	Secondary	Cycling time trial performance, power output/VO_2_	BP, plasma nitrite/nitrate	6.2 mmol beetroot juice	Nitrate-depleted beetroot juice	SBP −6 mmHg (*P* < 0.01), DBP (NS)	Clinic	Randomized, placebo-controlled, double-blind crossover (48–72 hr washout)	Acute 3 h
Lansley et al. [81]	Healthy, active (9 M) mean age 22 years	Secondary	O_2_ cost of walking/running (pulmonary VO_2_ and muscle PCr)	BP, HR, plasma nitrite/nitrate	6.2 mmol beetroot juice	Nitrate-depleted beetroot juice	SBP −5 mmHg (*P* < 0.01), DBP (NS)	Clinic	Randomized, placebo-controlled, double-blind crossover (10-day washout)	Chronic 6 days
Bailey et al. [82]	Healthy, active (7 M) mean age 28 years	Secondary	Muscle contractile efficiency (pulmonary VO_2_, PCr, ATP turnover)	BP, HR, plasma nitrite/nitrate	5.1 mmol/beetroot juice	Low-calorie blackcurrent cordial	SBP −5 mmHg (*P* < 0.05) DBP −2 mmHg (*P* < 0.05)	Clinic	Randomized, placebo-controlled, double-blind crossover (10-day washout)	Chronic 6 days
Kapil et al. study 1 [44]	Healthy *n* = 21 (M and F) mean age 23 years	Primary	BP	FMD, plasma nitrite/nitrate and cGMP	24 mmol KNO3	24 mmol KCI	SBP −9.4 mmHg (*P* < 0.001), DBP −6.0 mmHg (*P* < 0.001)	Clinic	Randomized, placebo-contolled, double-blind, crossover (7–28-day washout)	Acute 24 h
Kapil et al. study 2 [44]	Healthy *n* = 6 (M and F) mean age 29 years	Primary	BP	Plasma nitrite/nitrate	4 mmol or 12 mmol KNO_3_	n/a	SBP −9.4 mmHg (*P* < 0.001), DBP NS with 12 mmol dose	Clinic	Randomized, open-label crossover	Acute 3 h
Kapil et al. study 3 [44]	Healthy *n* = 9 (M and F) mean age 25 years	Primary	BP	Plasma nitrite/nitrate	5.6 mmol beetroot juice	Water	SBP −5.4 mmHg (*P* < 0.05), DBP (NS)	Clinic	Randomized, open-label crossover	Acute 3 h
Sobko et al. [60]	Healthy (10 M, 15 F) mean age 36 years	Primary	BP	Plasma and salivary nitrite/nitrate	0.3 mmol/kg/day Japanese traditional diet	Non-Japanese diet (0.06 mmol/kg/day)	SBP (NS), DBP −4.5 mmHg (*P <* 0.01)	Clinic	Randomized crossover (no washout period)	Chronic 10 days
Vanhatalo et al. [83]	Healthy, active (5 M, 3 F) mean age 29 years	Primary	BP (during exercise) and ventilatory/gas exchanged dynamics (e.g. VO_2_)	Plasma nitrite/nitrate	5.2 mmol/day beetroot juice	Low-calorie blackcurrent cordial	MAP −5.1 mmHg day 1–15 (*P* < 0.05)	Clinic	Balanced, randomized (10-day washout)	Chronic 15 days
Bailey et al. [84]	Healthy, active (8 M) mean age 26 years	Secondary	Exercise O_2_ cost and tollerance (e.g. HHb dynamics, VO_2_ and blood lactate)	BP, HR, plasma nitrite/nitrate	11.2 mmol/day beetroot juice	Low-calorie blackcurrent	SBP −8 mmHg (*P* < 0.01), DBP (NS)	Clinic	Randomized, placebo-contolled, double-blind crossover (10-day washout)	Chronic 6 days
Webb et al. study 1 [35]	Healthy (9 M, 4 F) mean age 26 years	Primary	BP	HR, plasma nitrite/nitrate	22.5 mmol beetroot juice	Water	SBP −10.4 mmHg (*P* < 0.01), DBP −8.1 mmHg (*P* < 0.01)	Clinic	Randomized, open-label crossover design	Acute 24 h
Larsen et al. [85]	Healthy, active (15 M, 2 F) mean age 24 years	Primary	BP	Plasma nitrite/nitrate	0.1 mmol/kg/day NaNO_3_	0.1 mmol/kg/day NaCI	SBP (NS), DBP −3.7 mmHg (*P* < 0.02)	Clinic	Randomized, placebo-controlled, double-blind, crossover (10-day washout)	Chronic 3 days

Table summarizing the methods and findings of clinical studies investigating the effects of inorganic nitrate on blood pressure in healthy volunteers. Study information includes participant information (*M* male, *F* female), primary and secondary study end points (*BP* blood pressure, *FMD* flow-mediated dilatation, *PWV* pulse wave velocity), nitrate and placebo sources and doses, summary of effects on systolic (SBP), diastolic (DBP) and mean arterial (MAP) blood pressure, blood pressure method of measurement (clinic, home or ambulatory (ABPM) blood pressure monitoring), study design and intervention length (acute or chronic). Numbers for referencing purposes are found in [*square brackets*]

**Table 2:** The effects of dietary inorganic nitrate on blood pressure in patient population studies

Author	Participants	BP primary or secondary endpoint	Primary endpoint	Secondary endpoint	Nitrate source/dose	Placebo	Effect on BP	BP measurement method	Study design	Intervention length
Bondonno et al. [86•]	Hypertensive (10 M, 17 F) mean age 63 years	Primary	BP	HR, plasma and salivary nitrite/nitrate, urinary Na^+^, K^+^ and creatinine	5.5 mmol/day beetroot juice	Nitrate-depleted beetroot juice	No effect	ABPM and home BP	Randomized, double-blind, placebo-controlled, crossover (2-week washout)	Chronic 1 week
Kapil et al. [87•]	Hypertensive (64 F) mean age 56 years (ctl) and 58 years (NO3^−^)	Primary	BP	HR, FMD, PWV, plasma, salivary and urinary nitrite/nitrate, plasma cGMP	6.4 mmol/day beetroot juice	Nitrate-depleted beetroot juice	Clinic −7.7/2.4 mmHg (*P* < 0.050). 24-h ABPM −7.7/5.2 mmHg (*P* < 0.001 for both). Home −8/3.8 mmHg (*P* < 0.001, *P* < 0.01)	Clinic, ABPM and home BP	Randomized, double-blind, placebo-controlled, crossover (2-week run-in)	Chronic 4 weeks
Bondonno et al. [88]	Pre-hypertensives (12 M, 26 F) mean age 61 years	Primary	BP, PWV, augmentation index	HR, plasma and salivary nitrite/nitrate	6.45 mmol/day nitrate-rich vegetables	Low-nitrate vegetables	SBP (NS), DBP (NS)	Clinic, ABPM and home BP	Randomized, placebo-controlled, crossover (7-day washout)	Chronic 1 week
Jajja et al. [89•]	Overweight, older (12 M, 9 F) mean age 62 years	Primary	BP	Salivary and urinary nitrite/nitrate	4.84–6.45 mmol/day (conc beetroot juice)	Blackcurrant juice	Daily SBP −7.3 mmHg during final week (*P* = 0.02), DBP (NS)	Clinic, ABPM and home BP	2-arm, parallel, randomized clinical trial	Chronic 21 days
Ghosh et al. [70]	Grade 1 hypertensives *n* = 15 (M and F) mean age 53 years	Primary	BP, PWV	HR, Plasma nitrite/nitrate and cGMP	3.3 mmol beetroot juice	Water	SBP −11.2 mmHg (*P* < 0.001), BP −9.6 mmHg (*P* < 0.001)	Clinic	Randomized, open-label crossover	Acute 24 h
Gilchrist et al. [90]	Type 2 diabetics (18 M, 9 F) mean age 67 years	Primary	BP, microvascular endothelial function, isoglycemic clamp	Plasma nitirte/nitrate	7.5 mmol beetroot juice	Nitrate-depleted beetroot juice	No effect	ABPM	Randomized, double-blind, placebo-controlled, crossover (4-week washout)	Chronic 2 weeks
Kelly et al. [91•]	Older, (6 M, 6 F) mean age 64 years M, 63 years F	Primary	BP, O_2_ uptake kinetics, and muscle (e.g. PCr) and cognitive function	HR, plasma nitrite/nitrate	9.6 mmol/day (conc beetroot juice)	Nitrate-depleted beetroot	SBP −5 mmHg (*P* < 0.05), DBP −3 mmHg (*P* < 0.05)	Clinic	Randomized, double-blind, placebo-controlled, crossover (72-h washout)	Chronic 3 days
Kenjale et al. [92•]	Peripheral artery disease (4 M, 4 F) mean age 67 years	Secondary	Exercise performance (e.g. VO_2_ and muscle PCr)	BP, HR, Plasma nitrite/nitrate, vascular testing	9.1 mmol beetroot juice	Orange juice	SBP −18 mmHg (*P* < 0.05) 2-min post exercise, DBP −8 mmHg (*P* < 0.05) at rest and during exercise	Clinic	Randomized, double-blind, placebo-controlled, crossover (7–14-day washout)	Acute 4 h

Table summarizing the methods and findings of clinical studies investigating the effects of inorganic nitrate on blood pressure (BP) in various patient populations, including hypertensive, diabetic and peripheral artery disease patients as well as older and/or overweight individuals. Study information includes participant information (*M* male, *F* female), primary and secondary study end points (*BP* blood pressure, *FMD* flow-mediated dilatation, *PWV* pulse wave velocity), nitrate and placebo sources and doses, summary of effects on systolic (SBP) and diastolic (DBP) blood pressure, blood pressure method of measurement (clinic, home or ambulatory (ABPM) blood pressure monitoring), study design and intervention length (acute or chronic). Numbers for referencing purposes are found in [*square brackets*]

Whilst the earliest observation of nitrate supplementation lowering blood pressure dates back to 1927 [93], it is the more recent observations that have raised the intense interest currently in the possible use of dietary nitrate as an anti-hypertensive. This interest was initiated in 2006 when Larsen et al. first demonstrated that a single oral sodium nitrate load of 0.1 mmol/kg/day for 3 days reduces diastolic blood pressure by 3.7 mmHg (*P* = 0.002). Soon after this, in 2008, Webb et al. demonstrated that a single dose of 22.5 mmol nitrate, provided in 500 ml beetroot juice, was capable of acutely decreasing systolic and diastolic blood pressure by 10.4 mmHg (*P* < 0.01) and 8.1 mmHg (*P* < 0.01), respectively: an effect still evident 24 h after ingestion with blood pressure remaining ∼6 mmHg lower than at baseline. Since then, numerous studies have been undertaken, investigating acute and chronic effects on blood pressure (see Table 1) as well as demonstrating dose-dependency of these effects [44] and successfully using a variety of sources of nitrate supplementation. Importantly dose-ranging studies have suggested that in healthy volunteers, somewhere between 4–12 mmol of nitrate lies a threshold dose for blood pressure lowering in individuals with ‘healthy’ blood pressures [44], although some studies have demonstrated efficacy with lower doses (see Table 1).

Table 1 summarizes key information from clinical trials reporting effects of dietary nitrate supplementation on blood pressure of healthy volunteers. Although results from these studies vary in the degree of blood pressure improvement, as well as showing reductions in systolic blood pressure, diastolic blood pressure, or both, the general conclusion, as drawn by a recent meta-analysis by Siervo et al., is that dietary nitrate supplementation is associated with a significant reduction in systolic blood pressure (*P* < 0.001) [94•]. In the latter analysis, meta-regression found an association between daily inorganic nitrate intake and change in systolic blood pressure (*P* < 0.05). Whilst some studies have reported a significant decrease in diastolic blood pressure, in this meta-analysis the overall effect of nitrate on diastolic blood pressure was not significant.

### Patient Studies

Although reports of blood pressure reductions in healthy volunteers have been encouraging and highlight the potential of inorganic nitrate for reducing blood pressure and cardiovascular disease risk, it was only in 2013 when the effects of nitrate on blood pressure in higher risk patient populations began to be reported. In 2013, Ghosh et al. reported a significant reduction in blood pressure in drug naïve hypertensive patients (with average baseline blood pressures of 152/90 mmHg), with impressive and significant reductions in both systolic and diastolic blood pressure (11.2 mmHg (*P* < 0.001) and 9.6 mmHg (*P* < 0.001), respectively) following an acute dose of 3.3 mmol nitrate administered in beetroot juice. Two years later, results from a larger 4-week, randomized double-blind, placebo-controlled trial, published by same group, reported significant and sustained reductions in systolic and diastolic blood pressure measured in the clinic as well as using 24-h ambulatory blood pressure monitoring (ABPM) and home blood pressure measurements. Reductions following consumption of 6.4 mmol/day nitrate varied between 7.7–8.1 mmHg (systolic) and 2.4–5.2 mmHg (diastolic) using the different methods of measurement, but all reductions were statistically significant [87•]. In this study patients were stratified into two groups, half were classified as stage 1 and the other half were stage 2 not to target, meaning that their blood pressure was raised despite taking one or more anti-hypertensive medication. On average the stage two patients were on two medications. Analysis of this group separately from the stage 1 patients indicated blood pressure lowering activity in both cohorts with, if anything, greater efficacy in the stage 2 patients.

Whilst the above results are encouraging, there have also been reports failing to demonstrate activity in similar cohorts. Bondonno et al., in a cohort of individuals classified as ‘pre-hypertensive’, showed no blood pressure lowering with a dietary nitrate dose of 400 mg/day which equates to just below 6.5 mmol [88]. In 2015, the same group demonstrated no significant effect on blood pressure in stage 2 hypertensive patients using 7 mmol/day nitrate, despite successfully increasing plasma nitrite levels [86•]. The reason for this discrepancy between the studies is uncertain. However, the patients in the former study had baseline systolic blood pressure recruitment criteria of 120–139 mmHg range with average blood pressures at baseline of the cohort of ∼129/76 mmHg. In the second study blood pressure was well controlled in the treated hypertensives with baseline blood pressures of ∼128/74 mmHg. It is possible that the lack of efficacy, in both of these studies, might relate to the lower baseline blood pressures particularly when one considers that the blood pressure lowering activity of any single dose of anti-hypertensive increases with increasing baseline blood pressure [95]. It is worth noting that the blood pressure lowering effect of nitrate in healthy volunteers with baseline blood pressures, not dissimilar to these studies, was minimal with a dose of 12 mmol and absent with a dose of 4 mmol suggesting that perhaps with higher doses greater efficacy may have been evident.

Another group of patients apparently resistant to the blood pressure lowering activity of nitrate are type II diabetics. Gilchrist et al. found no reduction in 24-h ambulatory blood pressure in patients who received 7.5 mmol/day of nitrate in beetroot juice for 2 weeks, despite causing a significant rise in plasma nitrite [90]. It is noteworthy that baseline blood pressures in this cohort were ∼135/70 mmHg ABPM and thus, as with the studies mentioned above, this lower baseline pressure may have had an impact upon efficacy. In addition, the volunteers received their dose of dietary nitrate (beetroot juice) in the evening. Studies monitoring ambulatory blood pressure show blood pressure lowering effects predominantly occurring during the daytime, following morning ingestion. This difference between protocols may underlie the disparity in results. The authors of this paper did also suggest that since the individuals recruited had a number of comorbidities they were taking a range of different medications and it is possible that this might also have had an impact upon efficacy.

Despite this discrepancy, it is encouraging that in other high risk patient populations, nitrate has been used with success, lowering systolic blood pressure of older and overweight patients by 5 and 7 mmHg, respectively after ingesting ∼10 mmol (for 2 days) and ∼5.5 daily (for 3 weeks), respectively [89•, 91•]. Importantly, these patients had baseline blood pressures of 125/74 and 135/77 mmHg. However, perhaps a difference from the negative studies mentioned above is that higher doses of nitrate were administered in one case whilst in another the study duration was longer. In patients with peripheral artery disease (PAD), diastolic blood pressure was also reduced by 8 mmHg at rest and during exercise, after consuming a single dose of 9.1 mmol nitrate, although systolic blood pressure was unaffected. Interestingly, 2 min into exercise recovery, systolic blood pressure was 18 mmHg lower in the nitrate treated group [92•]. This surprising discovery suggests that although at rest systolic blood pressure was not affected, there appears to be some effect following periods of physical activity. With reduction of nitrite to NO known to be promoted in hypoxic conditions [96, 97], it may be that, in these PAD patients, bouts of ischemia during exercise promote the reduction of plasma nitrite to NO resulting in the post-exercise reductions in blood pressure observed.

In sum, the beneficial effects of nitrate supplementation upon blood pressure are not limited to healthy individuals, but extend to (and are even enhanced in some) patient populations (see Table 2 for further information and references).

## Important Considerations

### Antibacterial Mouthwash and the Enterosalivary Circuit

There are now numerous reports supporting the critical role of the enterosalivary circuit in the effects of nitrate on blood pressure. In 2008 Govoni et al. demonstrated that in participants using an antibacterial mouthwash, to eliminate bacterial conversion of nitrate to nitrite selectively within the oral cavity, rises in circulating nitrite in response to an oral nitrate load are prevented [36]. Furthermore, in 2008, Webb et al. demonstrated in healthy volunteers that if recruits were prevented from swallowing their saliva for 3 h following an acute dose of nitrate, then the expected drop in blood pressure was no longer observed [35]. More recently, Kapil et al. demonstrated that not only is the enterosalivary circuit critical in blood pressure responses to acute and chronic dosing with dietary nitrate, but it is also important in normal physiological blood pressure control. In the absence of any dietary nitrate intervention, daily use of an antiseptic mouthwash caused a decrease in salivary and plasma nitrite levels of healthy volunteers by 90 and 25 %, respectively, an effect associated with an average increase in blood pressure of 3.5 mmHg [37•]. Evidence suggests that ∼31 % of the UK population regularly uses mouthwash [98] and nearer 60 % in the US [99]. The potential cardiovascular implications of such widespread daily use of mouthwash is therefore somewhat concerning, particularly in western society where a multitude of other cardiovascular risk factors are already highly prevalent. Whilst the use of mouthwash is in some cases a necessity, a more balanced view of the risks and benefits should certainly be considered following these findings with the aid of larger prospective clinical trials.

### Nitrate Sources and Placebos

As mentioned previously, various distinct sources have been tested in clinical studies investigating the effects of nitrate on blood pressure, including nitrate capsules and various forms of food supplementation, from spinach to beetroot juice and even nitrate enriched bread products (see Tables 1 and 2).

In recent years however, beetroot juice has been particularly popular as a convenient way to deliver a controlled dose of inorganic nitrate, both using concentrated shots and traditional beetroot juice drinks. However, it is worth noting that a recent study by Jajja et al. showed it is important for investigators to quantitatively assess the concentration of nitrate in their beetroot juices to confirm levels reported by the commercial supplier. For example, in this paper, the supplier reported a nitrate dose of 300–400 mg per bottle, but Jajja et al. found a concentration of only 165 mg per bottle. Such variations may underlie some of the differences in outcome reported in the literature.

Also, initially in trials using beetroot juice, as shown in Tables 1 and 2, placebo controls consisted of water, blackcurrant juice, orange juice or rice milk. This is important to consider when interpreting results from these studies, and when designing future studies, as blood pressure has been reported to be significantly influenced by ‘placebo effect’ [100, 101]. Fortunately, recent development in the field of nitrate research has led to the production of a nitrate-depleted beetroot juice using an ion exchange system [102]. Using this process means nitrate can be removed from the juice, but the remaining nutrient/mineral composition, as well as the taste and appearance, remain largely unchanged [103]. This process means beetroot juice and nitrate-depleted beetroot juice offer a convenient way to provide dietary nitrate in double-blind, randomized clinical trials with appropriately matched treatments and placebos.

## Potential Future Directions and Implications

### Heart Failure

It is now quite clear that inorganic nitrate has the potential to reduce blood pressure in both healthy individuals and hypertensive patients. One major implication of this is that such reductions in blood pressure could help reduce prevalence of co-morbidities, particularly such as heart failure, since hypertension is the most common risk factor shared by patients with heart failure (preceding heart failure in 66–90 % of cases [104, 105]).

However, it is also possible that inorganic nitrate may be capable of moderating cardiac hypertrophy/heart failure directly. Constitutive NO has been identified as an important modulator of cardiac hypertrophy as chronic inhibition of NO synthase, using L-nitroarginine methyl ester (L-NAME), has been shown to significantly increase angiotensin II-induced cardiac fibrosis in rats [106]. Both nNOS and eNOS knockout mice also develop cardiac hypertrophy spontaneously, with double knockout mice expressing a more severe cardiac hypertrophy phenotype [107, 108]. In accord with these observations is the finding that in patients with heart failure, a genetic variant of eNOS, resulting in shortened enzyme half life, is associated with increased mortality [109]. Low-level expression of NO therefore appears to be protective and loss of constitutive NO generation, for example as a result of endothelial dysfunction, may result in the loss of a braking mechanism of cardiac hypertrophic pathways. By restoring the ‘lost’ NO through nitrate supplementation such protective pathways may be restored. In support of this possibility, using a mouse transaortic constriction model of heart failure, sodium nitrite has recently proved to preserve cardiac ejection fraction, limit left ventricle remodeling and attenuate brain natriuretic peptide levels [110].

In healthy individuals inorganic nitrate also improves mitochondrial efficiency [111] and increase exercise capacity [112] and likewise nitrate has recently been shown to improve muscle contractile function and exercise capacity of heart failure patients [113–115]. Such findings suggest that inorganic nitrate supplementation is likely to be well tolerated and even beneficial in this patient population. However, as of yet the direct effects on the heart have not been established, and the potential for modulation of heart failure development and progression have not yet been investigated. Animal models of cardiac hypertrophy and heart failure are therefore likely to help us understand whether or not nitrate/nitrite/NO has a direct effect on cardiovascular remodeling and performance in addition to the beneficial effects on mitochondrial oxidative function, which have already been observed.

### Dialysis

Another patient population that could also potentially benefit from dietary nitrate research is dialysis patients. The importance of nitrate in cardiovascular health and blood pressure homeostasis has recently helped further understanding regarding the increased risk of cardiovascular-associated death observed in these patients. Little was previously understood about the mechanism(s) behind this phenomenon, but in 2013 it was found that the dialysis process reduced plasma nitrite levels by 57 % and nitrate by 84 %, likely resulting in a decrease in NO production and having negative effects on cardiovascular homeostasis [116]. Such a finding is likely to help, at least in part, explain the cardiovascular complications suffered by dialysis patients and importantly, results from further studies assessing the impact of dietary nitrate on cardiovascular outcome for dialysis patients would be of value.

## Conclusions

In conclusion, inorganic nitrate has proven time and again to be effective in reducing blood pressure in healthy individuals, and largely successful in reducing blood pressure in hypertensive patients. Importantly, these effects on blood pressure are dose-dependent, rely on the intact enterosalivary circuit (and can therefore be prevented by use of antibacterial mouthwash), are not susceptible to tachyphylaxis and are relatively consistent despite using different nitrate sources (capsule, beetroot juice, vegetables and bread). Thus, dietary nitrate offers a potentially cheap and effective way to help reduce blood pressure in hypertensives. In addition, dietary nitrate could also potentially offer options to improve cardiovascular outcome in other patient groups such as dialysis and heart failure patients, particularly since, in addition to blood pressure lowering, nitrate has been shown to be beneficial in improving exercise performance, tolerance and mitochondrial function. Although the effects (or perhaps lack of) in certain hypertensive patient subgroups, such as diabetics, remain to be further understood, in a large proportion of the general population, a diet rich in fruit and vegetables, or an alternative source of nitrate supplementation such as beetroot juice, is likely to be of significant benefit to cardiovascular health.
